# Chronic hand eczema - self-management and prognosis: a study protocol for a randomised clinical trial

**DOI:** 10.1186/1471-5945-12-6

**Published:** 2012-06-12

**Authors:** Annette Mollerup, Niels Kren Veien, Jeanne Duus Johansen

**Affiliations:** 1 National Allergy Research Centre, Copenhagen University Hospital Gentofte, Gentofte, Denmark; 2 The Dermatological practice at Vesterbro, Aalborg, Denmark

**Keywords:** Chronic hand eczema, Protocol for Self-management intervention, Randomised clinical trial, Patient counselling, The Healthy Skin Clinic, Nurse-led consultation

## Abstract

**Background:**

Hand eczema has a one-year prevalence of approximately 10 % in the general Danish population. Often the disease becomes chronic with numerous implications for the individual’s daily life, occupation and quality of life. However, no guidelines of self-management recommendations beyond the acute stage are given. Self-management of the disease is pivotal and involves self-monitoring of the condition, medication adherence, and preventive behaviour. Interventions best to support the individual in this ongoing process need to be developed.

**Methods/design:**

This paper describes the design of a randomised clinical trial to test a newly developed intervention of individual counselling versus conventional information. 300 patients consecutively referred to dermatologic treatment at two different settings are individually randomised to either the intervention programme, named ‘The Healthy Skin Clinic’ or to the control group. Block-wise randomisation according to setting and gender is carried out.

The intervention offers a tool for self-monitoring; basic and specific individual counselling; the possibility of asynchronous communication with the intervention team; and an electronic patient dialogue forum. Primary outcome variable is objective assessment of the hand eczema severity performed at baseline prior to randomisation, and repeated at six months follow-up. Secondary outcome variables are dermatology related life quality and perceived global burden of disease.

**Discussion:**

The trial aims at evaluating a newly developed guidance programme which is expected to support self-management of patients referred to dermatology treatment due to chronic hand eczema. The design of the protocol is pragmatic with blinding of neither participants nor the investigator. Thus, in the interpretation of the results, the investigator takes into account effects that may be attributed to actors of the interventions rather than the intervention per se as well of potential observer bias. Inclusion criterions are wide in order to increase transferability of the results.

**Trial registration:**

The trial is registered in ClinicalTrials.Gov with registration number NCT01482663.

## Background

Hand eczema is a pruritic inflammatory skin disease characterised in the acute phase by erythema, oedema and sometimes vesicles, while in the chronic state the skin changes are dominated by infiltration, scaling and fissures, which may cause pain. The disease is common [1]; in a recent Danish study a one-year prevalence of 14% was found, of which 23% had severe or moderate eczema [2]. Hand eczema is not a uniform disease as it exists in a continuum of severity with variations in morphology. Aetiologically, hand eczema may be due to irritant contact dermatitis, which is most prevalent followed by allergic contact dermatitis and atopic hand eczema [3]. Multi-causality is frequent and in order to provide adequate treatment and tertiary prevention aetiological factors need to be determined [4].

Hand eczema often has a chronic course with symptoms persisting 10–15 years after onset [3],5] and with psychosocial consequences such as long term sick leave, involuntary job rotation or early retirement [5]. Furthermore, quality of life has been shown to correlate negatively to the severity of the disease [4,6]. No consensual definition of chronic hand eczema exists although some authors have used the definition of hand eczema which persists throughout more than three months or which reoccurs twice or more within a 12 month time frame [7]. Chronic hand eczema typically has a dynamic course with intermittent eruptions of eczema [4]. A guideline for the overall treatment principles for hand eczema has been proposed by the Danish Contact Dermatitis Group [4]. However, although chronic hand eczema affects numerous functions in daily life, no specific recommendations as to self-manage the disease beyond the acute stage are given. Patients’ knowledge of their diagnosis may determine their prognosis [8]. Also, medical treatment of hand eczema is often prolonged and should be accompanied by skin protection and skin care measures [9]. However, some evidence based recommendations of skin protection may be experienced as complex [10]. Among dermatological patients it is estimated that 34–45% do not comply with instructions regarding medical treatment [11,12]. This may also depend on the satisfaction with and an overall experience of effectiveness of the treatment [13]. The patient’s self-management in the course is pivotal as it applies both to handling the acute eczema and to the continuous preventive behaviour, necessary to avoid relapses of the disease. Yet, evidence related to self-management of hand eczema is poor. In chronic illness generally, interventions that aim at increasing medication adherence, and thus indirectly the patient’s self-management, are most effective when intervening in several dimensions [14,15]. Better methods to support self-management of patients with chronic hand eczema may potentially improve the prognosis of a disease that is both a burden to the individual and to society. Hand eczema is often caused or aggravated by occupational exposures such as wet work, food handling, cutting oils etc. In several studies, interventions aiming at secondary prevention of occupational irritant hand eczema have been examined [16]. Comparisons have been made regarding the usage of protective equipment versus the usage of skin care products [17]. Also, the efficiency of skin care programmes including an educational element has been examined. Both skin protection behaviour and objectively assessed symptoms were beneficially impacted in a group of health care personnel [18,19] and among employees in the food care industry [20,21]. However in general, interventional prevention trials have had methodological weaknesses and insufficient dimensions in terms of power [16,18] and only few studies have investigated skin protection programmes as a tertiary preventive measure. In Germany, a combined health educational and health psychological intervention i.e. Tertiary Individual Prevention has been found to significantly increase important factors related to skin protection behaviour [22]. This programme requires three weeks of hospitalisation based upon employer-funded health insurance. Hence, these results are not easily transferred to a Danish health care system in which chronic hand eczema is predominantly treated in an outpatient setting. The current randomised controlled trial evaluates the effectiveness of a newly developed nurse-led counselling programme, based upon individual needs and resources, compared to conventional patient information given at medical consultations.

### Theoretical framework and regimen rationale

As aforementioned, chronic hand eczema is characterised by eczematous eruptions occurring in active phases of the disease. Medical treatment, primarily the usage of topical corticosteroids, is required during these eruptions. Periods between eruptions involve restitution of the skin and prevention of future eruptions in the context of daily life when the disease solely is handled as the individual’s self-management. Throughout the course, the individual may adapt to the condition in several dimensions. The progress in disease severity may lead to more apparent symptoms thus influencing the individual’s self-monitoring of the condition. Also, more severe eruptions of eczema may bring about increased involvement from health care professionals. Finally, the prolonged time frame and the repeated relapses can promote changes in the individual’s ability to cope and act upon disease-related problems based on experiences and required knowledge. These patterns of adaptation need to be considered in the support of patients’ self-management.

## Methods/design

### Objectives

The bulk of evidence mostly implies standardised intervention within specific occupational sectors that in particular dispose to hand eczema through wet work. However, in clinical dermatology practice the target group is highly heterogenic involving individuals in different phases of life and living under various conditions all of which may influence their self-management. A need for more individualised action plans and guidance yet still feasible in an institutionalised context has been identified.

The aim of the trial ‘Chronic Hand Eczema - self-management and prognosis’ is to evaluate if a newly developed guidance programme bring about a better prognosis of the disease at six months follow-up in comparison to conventional information.

### Hypotheses

It is hypothesised that the intervention may support the patients’ self-management to a degree that improvement in objectively assessed severity of hand eczema is measurable at a group level at six months follow-up compared to that of a control group. Also, improvement in subjectively assessed severity of disease and quality of life is expected.

### Empirical design

The hypotheses are tested through the conduct of a prospective randomised controlled trial having a pragmatic design [23]. The study is a multicenter trial as the setting is an outpatient clinic in a university hospital within the metropolitan area of the Danish capital, Copenhagen (setting A), and a private dermatology practice in Aalborg in northern Jutland, Denmark (setting B). For the purpose of this paper both settings are referred to as ‘the dermatologist’.

### Participants

Consecutive inclusion to the trial is performed. As soon as an individual is referred to treatment at the dermatologist due to ‘hand eczema’, an information letter is posted inviting the patient to participate in the trial. At inclusion, i.e. at first medical consultation at the dermatologist, signed, written informed consent is obtained. An objective evaluation of the present hand eczema is performed prior to an individual randomisation to either conventional information or to guidance according to the intervention. To enhance external validity of the trial, inclusion criterions are wide.

### Inclusion criterions

▪ Patients aged between 18–70 years (hand eczema is frequently work related and individuals older than 70 years may in general have a different pattern of exposure, which has to be considered at inclusion)

▪ A clinical diagnosis of hand eczema given by the dermatologist at the first consultation

▪ Signed, written informed consent to participate

### Exclusion criterions

▪ Patients in need of admittance to the dermatological in-hospital ward at the time of inclusion or during the follow up period

▪ Patients who cannot fill out a questionnaire in Danish due to language difficulties or to a physical or psychological handicap

### Randomisation

Patients, who fulfil the inclusion criterions, are included consecutively in the trial depending of time of their referral to the dermatologist. A baseline objective evaluation of hand eczema severity is carried out prior to the individual randomisation, which is performed centrally at the National Allergy Research Centre using a computer-generated algorithm unknown to the investigator. The patients are allocated to the intervention group, or conventional information in the control group. The percentage of patients allocated to each group is approximately 50:50. Randomisation is stratified by blocks according to setting and gender, the latter because of a gender ratio of hand eczema, as women have the double risk of men of hand eczema. Also, gender has been shown to impact both performed self-management [24] and ‘prescribed’ self-management [25].

### The trial flow

**Figure 1:**
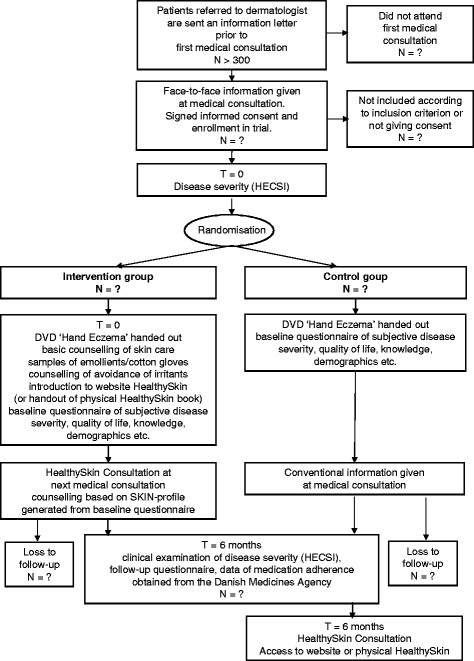
Trial flow chart.

When included in the trial, a baseline questionnaire is handed to the participant. Evidence from randomised trials suggests that in questionnaire design, ‘shorter is better’ [26]. Thus, attempts have been made to make the baseline questionnaire as short as possible. However, given the conceptual broadness of ‘self-management’, the questionnaire compiles items covering exposures, susceptibility, knowledge, medical adherence and self-efficacy. Immediately after randomisation, an initial basic counselling is given to participants in the intervention group. An appointment of the subsequent nurse-led consultation is offered to be conveniently executed in timely relation to the next medical consultation. Also, the patient is offered to choose between patient self-management books of either physical or electronic form. The trial flow is displayed in Figure 1.

### Trial intervention

The trial aims at intervening multi modally through initiatives which separately may be expected to support patient self-management and to be beneficial to the hand eczema prognosis [10,13,15,27-29]. The intervention is named The Healthy Skin Clinic in order to emphasise that self-management of hand eczema is more than medication adherence thus also involves health promotion. The Healthy Skin Clinic complies with usual clinical practice aiming to provide the patient with a systematic, yet individually, based counselling programme including a support module. The Healthy Skin Clinic is maintained by experienced nurses within the dermatological specialty along with the investigator (setting A), or by an experienced health care assistant supervised by the investigator and a dermatologist (setting B). A high level of team communication is conducted in order to provide commonality of the intervention.

The intervention is founded upon three concurrent elements. Firstly, a SKIN-profile is generated by responses from the baseline questionnaire. The profile is descriptive of the patient regarding susceptibility; knowledge; co-morbidity; social support; and necessary precautions i.e. allergies and aggravating irritant factors. One significant purpose of generating the profile is to clarify the multifactorial aetiology of (the persistence of) chronic hand eczema thus direct focus to the relevant areas in the following nurse-led consultation.

Secondly, a patient self-management book is offered. The book is a dynamic tool, which is launched in an electronic form, i.e. a secure website. Patients, with no possibilities of using information technology, will be handed over a physical book containing the same features. The patient self-management book contains information material which is tailored to the SKIN-profile. In addition, tools for self-monitoring of the disease are offered with the purpose of encouraging the patient to take a more proactive role in the course.

**Figure 2:**
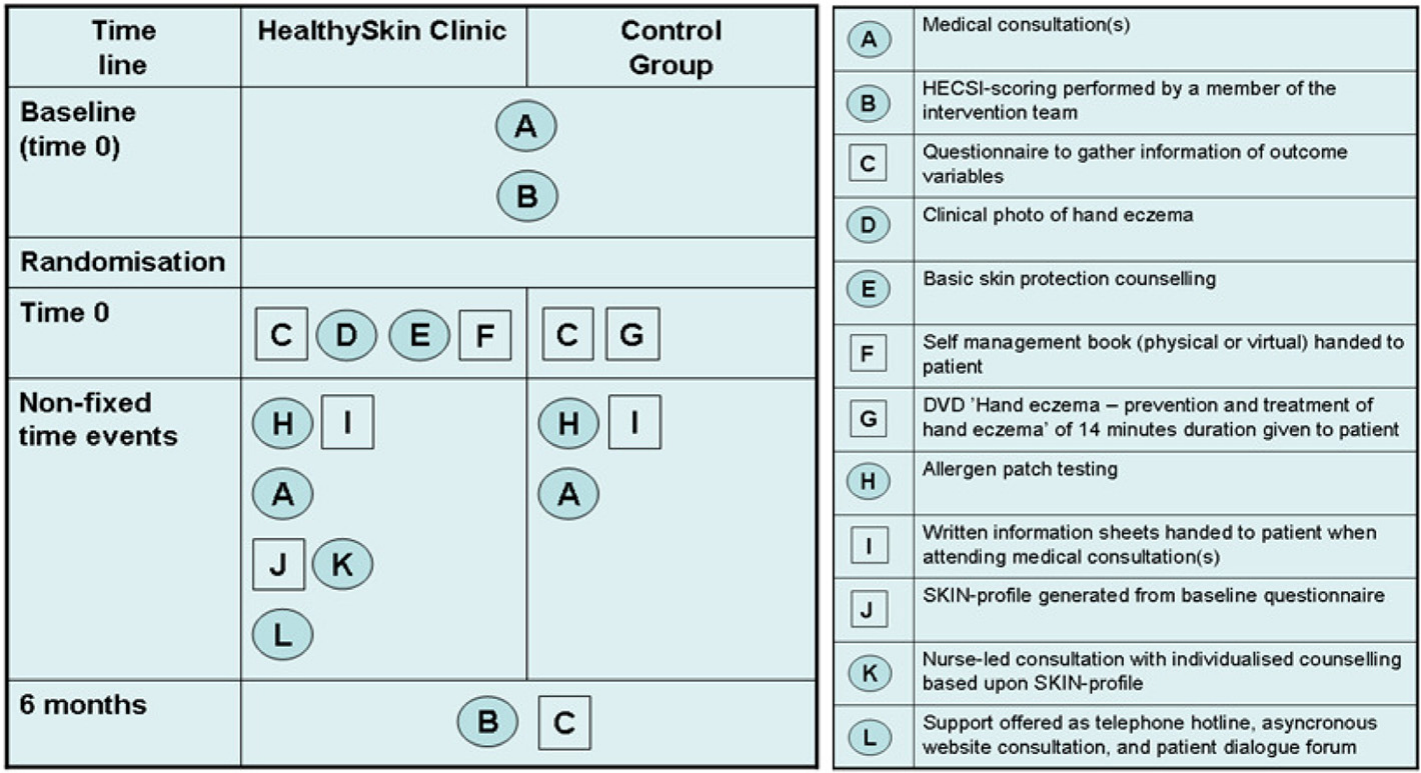
**Graphical depictions of interventions in the CHE-trial.** Components are regarded either as objects (represented by squares) or as activities (represented by circles) [30].

Finally, in addition to the nurse-led consultation, support to self-management through asynchronous communication and networking is offered. The online users may facilitate contact to either the intervention team or to other trial participants by use of the website. To the users of the physical self-management book a telephone based hotline is offered. An overall graphical depiction of the Healthy Skin Clinic intervention is presented in Figure 2 as this is a way proposed to clarify the content of complex interventions [30].

### Conventional information

At present in Denmark, no standards or guidelines as to information and guidance of patients with chronic hand eczema exist. The patients in the control group will receive conventional information, i.e. written information sheets, in relation to medical consultations and allergy tests.

### Measurement

Due to difficulties of measuring health behaviour, interventional efficacy is to be assessed by the clinical impact presumably brought about by the altered behaviour [27]. Hence, primary outcome variable is objectively assessed disease severity.

At the inclusion point, a baseline investigation of both control and intervention group is performed while collecting data demographics, medical history and predisposing factors of eczema disease, as well as information of occupational and leisure time related exposures. Wherever possible, questionnaires used in the trial are based on previously validated scales (see below). The variables will be re-evaluated at follow up after six months.

### Primary outcome

#### Objective severity of hand eczema

The primary outcome variable is the change from baseline (T_0_) to follow up in objectively assessed disease severity, which is evaluated clinically by a member of the intervention team. The assessment relies on the Hand Eczema Severity Index (HECSI) which is a validated scoring system developed to quantify severity of hand eczema. HECSI is a visual medical assessment and subjective symptoms of the patient, e.g. itch or pain are not included [31]. Inter-rater variability in the present trial will be evaluated by random checks where patients are scored twice independently and by different assessors.

### Secondary outcome

#### Quality of life

The patient’s subjective symptoms will be measured by Dermatology Life Quality Index (DLQI) and by parts of Impact of Skin diseases on Daily Life (ISDL). DLQI is a dermatology specific questionnaire of life quality which has been used globally since 1994. At the moment DLQI is the best validated instrument for measuring quality of life among dermatological patients [32]. However, DLQI is not reckoned to be adequate to evaluate the impact that chronic hand eczema has on daily life and being of the individual. ISDL is a newly developed multi dimensional instrument which only exists in an English version. So far, it has been validated to patients suffering from psoriasis and atopic dermatitis [33]. It includes questions of itching and scratching which both are frequent phenomena also when it comes to hand eczema and which is not noticeably covered by DLQI [33]. In addition, a self constructed Visual Analogue Scale (VAS) of perceived global burden of disease will be used.

#### Self-rated health status

As disease specific life quality presumably will be influenced by co-morbidity and life quality in general, a measurement of self-rated health will be conducted by use of the frequently used generic Health Status Questionnaire, Short Form (SF-36).

#### Patient competencies

Several factors might affect the prognosis of chronic hand eczema. These are included in the questionnaire i.e. literacy; motivation for medication adherence and the patient’s knowledge of causative exposure and management of hand eczema [11,13-15,34].

Literacy is evaluated from questions of the highest obtained education; Single-Item Literacy Screener; and self-rated reading proficiency [35-37]. Moreover, subjective action competence i.e. self-efficacy has been shown to determine changes in health behaviour [38]. Self-efficacy is measured at baseline through the Danish Version of the General Self-Efficacy Scale. This is an instrument which addresses self-efficacy as a concept related to personality traits [39]. Medication adherence will be evaluated by use of a Danish version of The Medication Adherence Report Scale (DMARS-4). This is a generic scale as yet solely validated to cancer patients in relation to their analgesic treatment [40]. In addition the two group’s primary medication adherence at follow up will be evaluated by gathering of register data from the Danish Medicines Agency [12]. Assessment of the patients’ own knowledge of exposures and management of their hand eczema will be carried out by use of parts of Nordic Occupational Skin Questionnaire (NOSQ-2002) [41]. This questionnaire is already available in a Danish version. Finally, the investigator has added a supplement of self-constructed questions regarding skin protection behaviour at follow-up.

#### Sick leave

Information of self-reported sick leave related to hand eczema both at baseline and at follow up will be obtained through the questionnaire.

#### Possible confounders

Several factors might affect self-management capabilities and the prognosis of disease thus are included in the questionnaire i.e. socio-economic status; perceived social support as well as depression and stress. In the data analysis a rough assessment of prevalent depression among participants is done by items included in the mental health sub-scale in SF-36 [42].

### Statistical plan and data analysis sample size estimation

Studies examining patient compliance by the use of intervention group and group of control patients are usually recommended to include a minimum of 60 participants in each group if an absolute difference in effect of 25% between groups is to be demonstrated at a probability of 80% [14]. Medication adherence is not easily measured in particular when it comes to dynamic conditions like eczema with treatment being a combination of a topical pharmaceutical and preventive behaviour [43]. Also, power calculation is hampered by uncertainty of whether an individual action plan may promote a clinically improvement when under the influence of exogenous factors related to the patient. In a previous study that used HECSI-score as outcome variable an average score of 11.2 was detected at six months follow-up compared to an average score of 19.9 at baseline (n = 366) [44]. With reference to this follow-up score in the control group and assuming an improvement of 30% due to the intervention, a power calculation carry out a sample size need of at least 87 patients in each group. In details, the sample size is calculated based upon the following values at six months follow-up along with estimated standard deviations: Control group mean-HECSI 11 (SD 9.0) and Intervention group mean-HECSI 7.7 (SD 6.0). Applying an alpha error level at 5%; and a beta error level at 90%, the use of 2-samples average data input bring forward a need of 87 participants in each group to detect a difference (http:/www.ddsresearch.com/toolkit/sscalc/size_a2.asp).

With a 15% excess of the number due to non-parametric variables and with caution due to the heterogeneities of the sample, and to the probability of dropouts, it has been assessed that inclusion of 150 patients in each group may be sufficient to show the anticipated effect of the intervention in question. No studies with estimated sample sizes based on ‘the minimal clinically importan difference’ of life quality measures have been identified. However, when using SF-36 as efficacy variable a minimum of 71 patients in each group are requested in order to demonstrate an achieved effect size of 0.5 [45]. Others propose a minimum of 80 in each group in interventional studies, targeting changes in health behaviour [46]. Hence, both figures are within the range of the present estimated sample size.

### Statistical methods

All data obtained at baseline will be given a coded ID-number before they are used in further statistical analyses. After double data entry and with the guidance of a statistician, statistical analysis will be performed in SPSS. Stratification for relevant confounders will be performed and descriptive statistics as well as multivariate analyses will be conducted. Ordinal scaled data will be evaluated as to a Gaussian distribution after which between-group differences will be tested in accordance by the use of *t*-test or non-parametric test. Dichotomous efficacy variables are tested by use of chi^2^ test or exact test in case of small numbers. The significance level will be a p-value of less than 0.05.

The final number of patients included in the trial will be reported and intention-to-treat analyses are to be performed meaning all participants will be included in the analysis irrespective of compliance with the intervention protocol. However, an additional per-protocol analysis will also be conducted. Data from patients who refused to participate, dropouts, and loss-to-follow-up participants will be entered to an analysis based upon demographics in order to consider selection bias when interpreting the results. The presentation of the trial results will be according to the CONSORT statement to Trials of Nonpharmacologic Treatment [47].

### Ethical considerations

The trial protocol has been approved by the Danish Data Protection Agency as well as the regional ethics committee (registration number H-2-2011-007). All data gathered will be coded in order to secure anonymity of the patient. Data are filed in secure drives and servers, and if physical i.e. questionnaires data, stored in secured archives thus only accessible to the investigator.

### Risk/benefits

The intervention of the trial is non-pharmaceutical thus potential risks relate to eventual wrongly given information and advise about skin care and skin protection. It is expected that misinformation will be hypothetical. The patients in the intervention group are expected to gain from the Healthy Skin Clinic in a way that makes them more knowledgeable and confident of their eczematous disease.

At present in Denmark, no standards or guidelines as to information and guidance to patients with chronic hand eczema exist. The patients in the control group will receive conventional information, i.e. written information sheets, in relation to medical consultations and allergy tests. Hence, they are not deprived from any usual care. By the end of the trial, participants in the control group are offered counselling given analogous to the Healthy Skin Clinic.

### Informed consent

By giving the signed, informed consent, participants accept to be informed of 1) the aim of the trial, 2) the voluntariness of their participation, 3) the fact that no consequences to the individual will occur as to continuous treatment if they choose to withdraw their consent, and 4) that all data concerning their health and daily life is to be used only with anonymity.

### Trial conduct

The trial will be conducted in compliance with the protocol, and no deviations from the protocol will be implemented without the prior review and approval of the regulatory authorities i.e. the Danish Data Protection Agency and the regional ethics committee.

### Blinding

The trial complies with usual clinical practice and the involved health professional actors will be the investigator and the members of the intervention team. It has been judged not to be feasible to maintain a fully blinded design throughout the trial. An effort is made to prevent performance bias by making the HECSI-scoring prior to randomisation. The HECSI scores are then removed from the participants’ data file to make the outcome assessment as blinded as possible. Also, when measuring primary outcome at six months follow-up, attempts are made to randomly allocate the member of the team, who does the HECSI-scoring. Hence, the participant may have been included by one member of the team; have been counselled by another; and have had final outcome assessed by a third member of the intervention team.

### Trial duration and discontinuation of individual participants

Duration of patients’ participation will be until follow-up six months after inclusion. It may be that the patient is discharged from treatment prior to date of completion or that dermatological treatment is necessary for a longer period than six months. However, outcome variables will be evaluated at six months follow up and no further intervention will be offered. The participants may continue to use the newly developed website, which is hosted and maintained by the National Allergy Research Centre. This includes access to information; the self-monitoring tool i.e. a personal log as well as the patient dialogue forum.

## Discussion

In this paper the protocol of a large randomised controlled trial in the clinical field of Dermatology is presented. The trial aims at evaluating a newly developed guidance programme which is expected to support self-management of patients referred to dermatology treatment due to chronic hand eczema. The design of the protocol is pragmatic and data analysis will be conducted with blinding of neither participants nor the investigator. Thus, potential observer bias may be a risk in the interpretation of the results. However, allocation to the two arms in the trial i.e. the intervention group and the control group is done by randomisation performed after the baseline assessment of the primary outcome variable. Other efforts are done in order to increase rigor of the trial. In the follow-up assessment of outcome variables, attempts are made ‘to be oblivious of’ the severity scores at baseline by deliberate removal of these sheets from the data files. Furthermore, the randomness inherent in the process of being a team of assessors both at baseline and at follow-up may prevent observer bias.

Finally, in the interpretation of the results, the investigator takes into account effects that may be attributed to actors of the interventions rather than the intervention per se [47,48].

## Competing interests

The authors declare that they have no competing interests.

## Authors’ contributions

AM and JDH are responsible for the overall design of the trial. AM is responsible for the protocol and has drafted the manuscript. AM is the principal investigator of the trial and project manager of the intervention team. NKV is situated at the setting in Aalborg, thus has contributed with minor protocol adjustments for this particular setting. Both JDH and NKV contribute throughout the trial by valuably reflections and discussions. All authors have read and approved the final manuscript.

## Pre-publication history

The pre-publication history for this paper can be accessed here:

http://www.biomedcentral.com/1471-5945/12/6/prepub
